# The Susceptibility Profiles of Human Peripheral Blood Cells to *Staphylococcus aureus* Cytotoxins

**DOI:** 10.3390/microorganisms13081817

**Published:** 2025-08-04

**Authors:** Tyler K. Nygaard, Jovanka M. Voyich

**Affiliations:** Department of Microbiology and Cell Biology, Montana State University, Bozeman, MT 59718, USA; jovanka@montana.edu

**Keywords:** *Staphylococcus aureus*, peripheral blood cell, cytotoxin, bicomponent leukocidin, neutrophils, erythrocytes, platelets, T cells, B cells, monocytes

## Abstract

*Staphylococcus aureus* is a Gram-positive bacterium that causes significant human morbidity and mortality. The capacity of *S. aureus* to cause disease is primarily attributed to an array of virulence factors produced by this pathogen that collectively overcome immune defenses and promote survival in a variety of host tissues. These include an arsenal of different cytotoxins that compromise plasma membrane integrity, with the specificity of each dependent upon the host organism and cell type. *S. aureus* encounters a variety of peripheral blood cell types during infection that play important roles in maintaining homeostasis and defending against microbial invasion, namely erythrocytes, thrombocytes, and leukocytes. *S. aureus* targets each of these cell types with specific cytotoxins to successfully establish disease. This review summarizes our current understanding of the susceptibility of different human peripheral blood cell types to each of these cytotoxins.

## 1. Introduction

*Staphylococcus aureus* (*S. aureus*) is a common Gram-positive bacterium that can colonize and infect a wide range of hosts. Though generally a benign commensal found in the anterior nares of approximately one third of the human population [1], this bacterium can also cause a spectrum of infections that range from superficial skin abscesses to life-threatening systemic disease [2]. Our capacity to treat these infections is complicated by the widespread acquisition of antibiotic resistance mechanisms. In addition, a vaccine to prevent *S. aureus* disease has been unsuccessful despite extensive efforts [3,4,5]. *S. aureus* remains a significant source of human morbidity and mortality worldwide [6], underscoring the need to further our understanding of *S. aureus* virulence to advance novel therapeutic strategies that limit pathogenesis.

The ability of *S. aureus* to infect numerous host tissues in a variety of organisms is largely attributed to an arsenal of diverse virulence factors that include multiple cytotoxins, host immunomodulatory proteins, and adhesins. Cytotoxins target host cells in a receptor-dependent or receptor-independent manner, compromising plasma membrane integrity to cause loss of function and lysis. Many excellent reviews detail the structure and function of each of these cytotoxins [7,8,9,10,11,12,13,14]. However, there have been no reviews that have focused on the susceptibility of human peripheral blood cells to these cytotoxins. Defining these susceptibility profiles will expand our understanding of how *S. aureus* specifically targets host cells to advance bacterial survival and pathogenesis.

Clinical *S. aureus* isolates express multiple cytotoxins known to be active against a variety of human cell types (Table 1). Perhaps the most characterized of these is α-hemolysin (Hla), which has been shown to recognize ADAM10 [15] and form a heptameric β-barrel pore composed of monomeric subunits on a wide range of target cells. The bicomponent leukocidins are a group of pore-forming cytotoxins that bind different host cell receptors and form an octameric β-barrel pore composed of dimeric subunits. These include γ-hemolysin A/B (HlgAB) that recognizes CCR2, CXCR1, CXCR2, and the Duffy antigen receptor for chemokines (DARC) [16,17]; γ-hemolysin C/B (HlgCB) that recognizes C5aR1, C5aR2 [16]; leukocidin E/D (LukED) that recognizes CCR5, CXCR1, CXCR2, and DARC [17,18,19]; leukocidin G/H (LukGH [20], also known as LukAB [21]) that recognizes CD11b and the voltage-gated hydrogen channel 1 (HVCN1) [22,23]; and the Panton-Valentine leukocidin (PVL) that recognizes C5aR1, C5aR2, and CD45 [24,25]. The phenol-soluble modulin peptides (PSMs) are small amphipathic peptides thought to act like detergents and interact with host cell membranes in a receptor-independent manner [14,26,27]. These include δ-toxin (Hld), the phenol-soluble modulin-α (PSMα) peptides, and the phenol-soluble modulin-β (PSMβ) peptides. Like the PSMs, β-toxin (Hlb) degrades plasma membranes but does not generate a true pore on susceptible host cells [7,28]. Membrane damage is generated by the sphingomyelinase activity of Hlb and may be mediated through a specific host cell receptor, though none have been identified to date [7]. Notably, the majority of *S. aureus* strains associated with humans have the Sa3Int prophage integrated into the gene encoding Hlb that deactivates expression of this cytotoxin [29,30,31,32,33,34,35,36,37,38,39,40,41,42,43,44,45]. The number and apparent redundancy of these cytotoxins has made it difficult to parse out the relative importance of each during different facets of human disease. Further, host specificity [7,8,10,13,46,47,48,49,50,51,52,53,54] has limited the utility of non-human cell lines and animal models of infection to understand the contribution of each cytotoxin to *S. aureus* pathogenesis in humans.

*S. aureus* gene expression in vivo is largely dictated by the concerted influence of 16 two-component regulatory systems that recognize environmental stimuli and alter gene expression in response [104,105,106]. Two of these, the accessory gene regulator (Agr) two-component system and the *S. aureus* exoprotein (Sae) two-component system, are recognized as major regulators of cytotoxin production. Agr is a quorum-sensing system that increases expression of all cytotoxins at high bacterial concentrations [107,108]. This two-component system primarily regulates gene expression in a post-transcriptional manner via the action of RNAIII [109,110] but also through transcriptional regulation of PSMs [111]. In contrast, Sae is thought to recognize neutrophil-associated stimuli and respond by upregulating the transcription of almost all the cytotoxins but not PSMs [112,113,114,115,116,117,118]. As the activity of two-component regulatory systems is largely driven by environmental cues, exclusive examination of cytotoxicity caused by purified proteins or *S. aureus* during in vitro growth may not accurately reflect the relative contribution of each cytotoxin to *S. aureus* virulence in vivo.

*S. aureus* encounters a variety of circulating peripheral blood cell types during infection, namely erythrocytes (red blood cells) that primarily maintain host tissue perfusion, thrombocytes (platelets) that play a key role in tissue repair, and leukocytes (neutrophils, T cells, B cells, monocytes, eosinophils, and basophils) that are a vital defense against microbial infection. The ability of *S. aureus* cytotoxins to compromise these cell types is essential for bacterial survival and dissemination in the healthy human host. This review summarizes our current understanding of the importance of each of these cytotoxins in causing lysis of different peripheral blood cells in humans.

## 2. Erythrocytes

Erythrocytes are considerably more abundant than other peripheral blood cell types, with approximately 4 to 6 million cells per microliter of adult human blood [119]. These anucleated cells are specialized to transport oxygen from the lungs to host tissue using hemoglobin and each contains approximately 270 million copies of this protein. Reversible binding of oxygen by hemoglobin is enabled by four iron-containing heme molecules. Iron is an essential metabolite for nearly all organisms and failure of bacteria to obtain iron in the host limits pathogenesis [120,121]. *S. aureus* acquires this important metabolite during infection following hemolysis of host erythrocytes and sequestration of released heme using the iron-regulated surface determinant (Isd) system [122,123]. Thus, a clear understanding of the specific cytotoxins produced by *S. aureus* that cause significant hemolysis of human erythrocytes will underscore virulence factors that are important for iron acquisition and consequent pathogenesis in humans.

Traditionally, hemolysis produced by *S. aureus* has been measured using sheep blood agar and rabbit erythrocyte lysis assays. However, it has been shown that the susceptibility of animal erythrocytes to *S. aureus* cytotoxins differs significantly from human erythrocytes [46,48,49,50] and agar is known to limit the hemolytic activity of HlgAB [48]. Recent investigations have helped to clarify which cytotoxins target human erythrocytes using assays that examine these cell types in solution. These studies have measured the hemolytic activity of purified proteins, extracellular factors produced by *S. aureus* deletion mutants during in vitro growth, and the effects of co-culturing these mutants with human erythrocytes.

Using purified proteins, it has been shown that strong hemolysis of human erythrocytes is caused by HlgAB and LukED, while HlgCB, Hla, PVL and LukGH produce limited or no hemolysis [17,75,77,78,124]. Interestingly, noncanonical pairing of purified HlgA with LukD was shown to exhibit more potent hemolytic activity than other bicomponent leukocidin pairs [54]. It has also been demonstrated that hemolysis of both HlgAB and LukED require erythrocyte expression of the Duffy antigen receptor for chemokines (DARC) [17], a chemokine receptor also used by *Plasmodium vivax* and *Plasmodium knowlesi* to invade erythrocytes during malarial pathogenesis [125,126]. DARC is not expressed by many individuals of African descent, reducing susceptibility to malaria as well as hemolysis caused by HlgAB and LukED. Others have also shown that purified PSMα1, PSMα2, PSMα3, PSMβ1, and Hld exhibit pronounced hemolytic activity against human erythrocytes while PSMα4 and PSMβ2 are also hemolytic but to a lesser extent [74,127]. In addition, synergy between purified PSMs and Hlb has been shown to enhance hemolysis, whereas all but one study indicate that Hlb alone is not hemolytic against human erythrocytes [72,74,102,128].

Research examining hemolysis of human erythrocytes in suspension by cytotoxins produced by *S. aureus* during growth has been more limited. A study using extracellular factors produced by *S. aureus* laboratory strain Newman during in vitro growth has shown that HlgA plays a major role in hemolysis of human erythrocytes [76]. However, this same study indicated that hemolysis caused by extracellular factors produced by the clinically relevant *S. aureus* strain USA300 was largely independent of HlgAB. This discrepancy can be explained by a point mutation in the histidine kinase sensor SaeS in strain Newman that induces constitutive activation of the Sae two-component system and consequent overexpression of numerous secreted virulence factors [117,129]. Others have demonstrated that transcription of *hlgAB* is highly upregulated following exposure of USA300 to human blood, suggesting that specific in vivo environmental stimuli are needed to trigger HlgAB mediated cytotoxicity caused by this strain. Indeed, it was shown that USA300 grown in the presence of human erythrocytes expressing DARC caused hemolysis that was dependent upon HlgA [17]. In contrast, others have used extracellular factors produced by *S. aureus* lacking PSMα to show that these peptides are a dominant factor causing hemolysis of human erythrocytes [60,102]. Collectively, these studies indicate that HlgAB, LukED, and PSMα peptides play major roles in causing hemolysis during *S. aureus* infection in humans. However, a comprehensive analysis directly comparing the contribution of these cytotoxins in causing hemolysis of human erythrocytes has not been performed.

## 3. Thrombocytes

Thrombocytes, or platelets, are essential for maintaining vascular integrity following host tissue injury. Platelets are anucleated cell fragments shed from megakaryocyte extensions in the bone marrow and are abundant in circulation, with approximately 150,000 to 450,000 platelets per microliter of adult human blood [119]. Platelets play a major role in primary hemostasis by adhering to the site of vascular injury, forming aggregates that plug the damaged vessel, and releasing factors that trigger thrombosis. In addition to maintaining vascular integrity, platelets also have important roles in limiting infection that include alerting the immune system to invading microbes, performing antimicrobial functions, and enhancing the adaptive immune response [130,131,132,133,134].

Clinical evidence that thrombocytopenia in patients with *S. aureus* bacteremia corresponds to increased mortality [58,135,136] indicates that platelets are an important defense against systemic *S. aureus* infection in humans. Platelets have been shown to bind and trap different bacteria including *S. aureus* to promote neutrophil phagocytosis [137], and antimicrobial peptides produced by human platelets kill *S. aureus* and induce extracellular trap formation by neutrophils [58,137,138]. However, *S. aureus* produces numerous virulence factors that enhance platelet aggregation and activation, including ClfA, ClfB, SpA, FnBPA, FnBPB, SSL5, and Hla [56,139,140,141,142,143,144,145], suggesting this pathogen manipulates platelet function to enhance survival in vivo [146]. In addition, *S. aureus* produces at least one cytotoxin, Hla, that lyses human platelets.

Investigations have demonstrated that purified Hla compromises the viability of human platelets [56,57,58] and *S. aureus* expression of this cytotoxin enhances platelet destruction during ex vivo infection [58]. Evidence also indicates Hla triggers apoptosis of human platelets [57]. In contrast, purified LukGH, LukED, or PVL did not influence platelet activation or viability [57]. These results are supported by the observation that resting platelets express ADAM10, the host cell receptor recognized by Hla, but not other host cell receptors recognized by *S. aureus* cytotoxins [147,148]. However, activated platelets can express both CD11b [149] and C5aR [150,151,152], indicating that certain in vivo conditions can induce platelet susceptibility to LukGH, HlgCB, and PVL. In addition, the impact of PSMs on human platelet integrity has not been examined. Collectively, these findings indicate that Hla plays a prominent role in causing lysis of human platelets, but further investigations are needed to conclusively determine the influence of other *S. aureus* cytotoxins on platelet viability.

## 4. Neutrophils

Neutrophils are the most common circulating leukocytes, with approximately 2500 to 8000 cells per microliter of adult human blood [119]. These granulocytes are relatively short-lived, with a half-life of around 7 hours in circulation. Neutrophils are a major defense against microbial invasion and play a particularly important role in preventing *S. aureus* disease [41,153]. Neutrophils in circulation are primed following detection of host- and/or microbe-derived signaling molecules released during infection and migrate to the site of distressed host tissue through a process referred to as extravasation [154]. Phagocytosis of invading microbes is primarily mediated by various Fc receptors on the neutrophil cell membrane that bind to immunoglobulins and complement components that have opsonized the microbial cell surface. Following phagocytosis, bacteria are normally terminated by an array of antimicrobial peptides and reactive oxygen species released within the phagosome.

In contrast to most bacteria, *S. aureus* can not only survive phagocytosis by expressing factors such as superoxide dismutase [155], catalase [156], and SPIN [157,158], but can also subsequently compromise neutrophil integrity and function via the production of cytotoxins [159,160]. When a substantial number of *S. aureus* is present in host tissue, such as an established *S. aureus* abscess, high concentrations of secreted cytotoxins can also preemptively strike incoming neutrophils to impede direct engagement with this pathogen. Neutrophils express all the receptors recognized by the receptor-dependent cytotoxins produced by *S. aureus* [161,162,163] and numerous investigations have shown these cells are susceptible to each. These studies have tested the susceptibility of human neutrophils to purified proteins, intoxication by extracellular factors produced by *S. aureus* cytotoxin deletion mutants, and cell destruction following exposure to these deletion mutants. However, the relative importance of each cytotoxin in the lysis of these important immune cells following initial inoculation, dissemination through the lymphatic and circulatory systems, and colonization of distal host tissue is not entirely understood.

**Figure 1:**
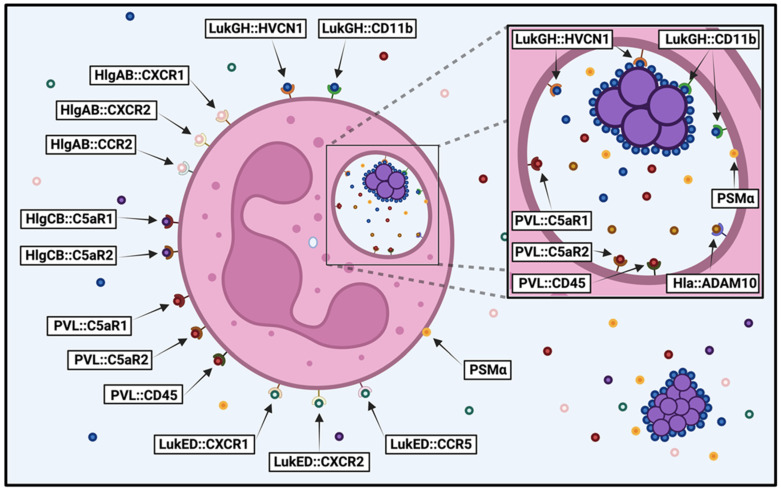
Susceptibility of human neutrophils to *S. aureus* cytotoxins. Diagram illustrating the *S. aureus* cytotoxins that target human neutrophils before and after phagocytosis, detailing the host cell receptors recognized by each cytotoxin (cytotoxin::host cell receptor). Image created in BioRender.

Purified HlgAB, HlgCB, LukED, LukGH, PVL, and PSMα peptides have been shown to cause plasma membrane permeability of primary human neutrophils [16,18,21,22,23,24,25,27,51,52,79,80,81,83,84,86,92,93,94,95,96,97,98,99,100]. Of these, purified HlgCB, LukGH, and PVL appear to have the most potent activity against these cell types [79,81]. Interestingly, non-canonical pairing of HlgCB and PVL appears to exhibit enhanced cytotoxicity [81]. Intoxication by extracellular proteins produced by *S. aureus* deletion mutants has indicated that secreted LukGH, HlgAB and/or HlgCB, PVL, and the PSMα peptides all play a role in compromising neutrophil plasma membrane permeability, with in vitro culture conditions and type of *S. aureus* strain largely dictating the relative importance of each [20,21,27,52,82,87,94]. Alternatively, studies measuring neutrophil integrity following exposure to live *S. aureus* have also shown that Hla, LukGH, PVL, and PSMα peptides all contribute to destruction of these immune cells to varying degrees that appear to be dependent upon the *S. aureus* strain examined [20,21,22,52,59,60,61,80,82,86,87,88,103]. Taken together, these studies indicate that most cytotoxins produced by *S. aureus* have the capacity to destroy human neutrophils and the context of intoxication influences their potency (Figure 1). 

Though numerous cytotoxins have been shown to target human neutrophils, which of these are most important for causing lysis of these important innate immune cells is not clear. To clarify the relative contribution of each cytotoxin to human neutrophil destruction, we recently used a library of deletion mutants in *S. aureus* strain USA300 to examine neutrophil lysis following intoxication by extracellular proteins and after phagocytosis [89]. We found that PVL played a dominant role in initially compromising neutrophil integrity caused by extracellular proteins produced by USA300 during growth in a variety of media. In contrast, LukGH was the primary cytotoxin causing neutrophil destruction immediately following phagocytosis. The different context-dependent cytotoxicity exhibited by these two apparently redundant bicomponent leukocidins can be explained by the unique location and structure of LukGH. There is only 26–40% sequence homology between LukGH and the other bicomponent leukocidins [20,21,164]. Unlike other cytotoxins, LukGH is expressed at high levels on the surface of *S. aureus* [20,165] and is preassembled in its activated form prior to engagement with neutrophils [166,167]. In conjunction with our results, and as previously speculated by others [7], this suggests that LukGH is poised on the surface of *S. aureus* to immediately engage with phagocytes upon direct contact. Thus, PVL appears to play a major role in lysing incoming neutrophils when *S. aureus* is established in host tissue and producing high concentrations of cytotoxins, while LukGH is the primary cause of lysis following direct contact with neutrophils and may be important for survival following initial inoculation or dissemination of this pathogen when the concentration of other cytotoxins is relatively low.

However, there are several caveats to our study. Cytotoxicity was only examined using *S. aureus* strain USA300 and substantial genetic diversity is found between different *S. aureus* isolates [168]. Notably, a single point mutation in the 5′ untranslated region of the *hlgCB* operon of USA300 minimizes translation of HlgC relative to HlgB [169]. Although neutrophil phagocytosis triggers significant alterations in *S. aureus* gene expression [159], other stimuli associated with infection of host tissue were largely lacking from this study [170]. For example, it has been shown that transcription of the *hlgABC* operon is immediately upregulated following exposure to human blood [82,171] and transcription of the *hlgABC* operon, *hla*, *lukED*, and *pvl* are upregulated during cutaneous infection in humans [172,173]. In addition, the activation state of neutrophils influences the expression levels of host cell receptors that are recognized by cytotoxins [162], suggesting the potency of these cytotoxins can vary in vivo under different conditions. Future studies that incorporate these factors may elucidate more profound roles for other cytotoxins in causing human neutrophil lysis.

## 5. T Cells

T cells are the most common circulating lymphocytes, with approximately 800 to 2500 cells per microliter of adult human blood [119], and are the primary constituents of the adaptive cellular immune response. T cells have been broadly classified as CD4^+^ helper T cells (Th cells) that direct the immune response through cytokine expression or CD8^+^ cytotoxic T cells that eliminate diseased host cells. Each T cell expresses a unique T cell receptor that induces cell activation and proliferation upon binding a specific antigen in conjunction with additional signals provided during antigen presentation. The distinct antigen-binding capacity of each T cell receptor on Th cells elicits a discrete cytokine expression profile directed towards specifically eliminating the source of that antigen [174]. Given the consistent failure to produce a *S. aureus* vaccine, the importance of T cells in protecting against *S. aureus* infection has gained appreciation in the last several decades [175,176,177,178].

*S. aureus* produces numerous virulence factors that specifically target T cell integrity and function, namely superantigenic proteins and cytotoxins. Superantigenic proteins bind directly to the T cell receptor on T cells and MHC Class II on antigen presenting cells to generate antigen-independent activation of approximately 20% of T cells, roughly 25,000-fold greater than a normal T cell response [179,180,181,182,183]. This produces robust inflammation and is thought to drown out an effective Th cell response that would otherwise limit pathogenesis. In addition to superantigens that manipulate T cell function, there are several *S. aureus* cytotoxins known to compromise T cell plasma membrane integrity.

Primary human T cells appear to be more resistant to *S. aureus* cytotoxicity than human neutrophils or monocytes [63,68]. T cells can express ADAM10 [184], CCR2 [185,186,187], CCR5 [185,187], CD11b [188,189], CD45 [190], CXCR1 [191,192,193], and C5aR [194,195] to varying degrees in vivo, indicating these lymphocytes are susceptible to intoxication by Hla, HlgAB, HlgCB, LukED, LukGH, and PVL. However, studies using purified proteins have shown that primary human T cells are susceptible to Hla [62,63,64,65,66,67] and LukED [19], but resistant to PVL [24,93] and other bicomponent leukocidins [79]. Others have shown that purified Hlb is cytotoxic against proliferating [28] but not resting human T cells [73]. Intoxication of human T cells with extracellular factors produced by an isogenic deletion mutant of Hla in *S. aureus* strain USA300 and incubation of this isolate with human T cells further demonstrated the importance of this pore-forming toxin for lysis of these lymphocytes [63]. Evidence indicates that T cells exposed to low concentrations of Hla form small pores that only allow passage of monovalent ions and trigger programmed cell death while high concentrations of Hla result in plasma membrane permeability of larger molecules and immediate cell lysis [63,66]. Other investigations using an antibody that neutralizes LukGH-mediated cytotoxicity indicated this bicomponent leukocidin does not target human T cells [80]. T cell expression of CCR2 [186,187], CCR5 [187], CD11b [188,189], CXCR1 [191,193], and C5aR [194] is influenced by activation state, suggesting that the potency of HlgAB, HlgCB, LukED, LukGH, and PVL against these cells may vary under different in vivo conditions. Though evidence indicates that Hla and LukED are the primary factors produced by *S. aureus* that cause human T cell lysis, a comprehensive analysis examining the relative contribution of these and other cytotoxins towards the destruction of these lymphocytes has not been performed.

## 6. B Cells

B cells produce immunoglobulins, or antibodies, that are the foundation of the adaptive humoral immune response. These lymphocytes are less abundant than T cells, with approximately 100 to 450 B cells per microliter of adult human blood [119]. Akin to T cells, each B cell possesses a unique B cell receptor that is composed of an immunoglobulin anchored to the cell membrane and associated with embedded accessory proteins. B cell activation, proliferation, and differentiation into antibody-producing plasma cells requires antigen binding to the B cell receptor and secondary signals generally provided by Th cells. Circulating antibodies not only bind to microbes to promote phagocytosis and inhibit adhesion but also bind to secreted microbial virulence factors to neutralize their activity. As such, B cells play an essential role in adaptive immunity against microbes.

The importance of B cells in preventing infections caused by *S. aureus* is not clear. Humans with deficiencies in B cell numbers or function are not more susceptible to *S. aureus* disease [5,196,197,198], suggesting that B cells are dispensable for preventing *S. aureus* pathogenesis. This might explain why the same strain of *S. aureus* can cause repeated infections in humans and vaccine efforts against this bacterium have universally failed. However, it is also possible that *S. aureus* is extremely adept at limiting an efficient adaptive humoral immune response. Indeed, *S. aureus* produces virulence factors that not only manipulate Th cell function to indirectly impede appropriate B cell activation, as described above, but also directly minimize the function of antibodies and B cells. These include the Staphylococcal protein A (SpA) and staphylococcal binder of immunoglobulin (Sbi) that both bind to human antibodies to inhibit their activity [199,200,201,202]. In addition, SpA is superantigenic against human B cells that express VH3-encoded immunoglobulin, causing widespread antigen-independent activation and subsequent apoptosis of these cells [203,204,205,206]. *S. aureus* also expresses cytotoxins that cause human B cell destruction, though these lymphocytes appear to be more resistant to *S. aureus* cytotoxicity than other peripheral blood cell types [63,68].

Given the repeated failures to produce an *S. aureus* vaccine and that B cell integrity is critical for an efficient adaptive humoral immune response, it is surprising that there have been few investigations that have examined the potency of *S. aureus* cytotoxins against human B cells. These lymphocytes express ADAM10 [207], CCR2 [186], and CD45 [190], indicating that they are theoretically susceptible to Hla, HlgAB, and PVL. However, currently published studies have only demonstrated that primary human B cells are susceptible to Hla. These have shown that purified Hla targets primary human B cells [62,63,65]. Further analysis of extracellular factors produced by an isogenic deletion mutant of Hla in USA300 as well as following infection of B cells with this mutant confirmed the importance of this cytotoxin for causing B cell lysis [63]. This study also demonstrated that Hla causes programmed cell death of B cells. However, it was shown that USA300 lacking Hla still causes B cell lysis [63], suggesting that other cytotoxins also contribute to destruction of these cell types. Other studies have indicated that human B cells are resistant to cytotoxicity caused by LukGH [80] or PVL [83,93] while the impact of PSMs on the viability of these lymphocytes has not been addressed. Collectively, these findings demonstrate that B cells are susceptible to Hla but also indicate that other cytotoxins produced by *S. aureus* target these important adaptive immune cells.

## 7. Monocytes

Monocytes are professional phagocytes that play important roles in both the innate and adaptive immune responses. These cells are less abundant in peripheral circulation relative to other leukocytes, with approximately 100 to 700 monocytes per microliter in adult human blood [119]. Though monocytes were traditionally thought to primarily differentiate into macrophages and dendritic cells following recruitment to host tissue, current evidence suggests that these antigen-presenting cells can also remain undifferentiated and exhibit both pro-inflammatory and anti-inflammatory characteristics [208]. Monocytes play an important role in limiting infection by recognizing microbial invaders and coordinating an appropriate immune response through cytokine signaling and antigen presentation.

Though monocytes likely play an important role in the immune response against *S. aureus* and numbers of these cells significantly increase during systemic *S. aureus* disease [209], evidence suggests that this pathogen can undermine the function of these leukocytes to further bacterial survival and dissemination [210,211,212]. Studies have shown that *S. aureus* can survive phagocytosis by primary human monocytes and monocyte-derived macrophages and it has been proposed that this furthers dissemination in the host [211,212,213]. Others have indicated that the cytokine response to live *S. aureus* during infection of human blood is suppressed relative to heat-killed *S. aureus* [210], *S. aureus* lacking the Sae two-component system [210], or *S. aureus* lacking Hla [68]. The mechanisms by which *S. aureus* manipulates monocyte function are not entirely clear, but cytotoxins have been implicated in this process [68,210,211].

Relative to other human leukocytes, evidence suggests monocytes are highly susceptible to cytotoxicity caused by *S. aureus* [63,68]. As with neutrophils, these cell types express all the receptors recognized by the receptor-dependent cytotoxins [214,215,216,217,218]. Indeed, studies have shown that purified Hla, Hlb, HlgAB, HlgCB, LukED, LukGH, and PVL can all cause lysis of primary human monocytes [16,19,23,24,62,63,73,75,83,90,93,101]. Infection assays using isogenic deletion mutants of LukGH and Hla confirmed the importance of these cytotoxins for causing monocyte lysis [63,68,90]. Though PSMs have not been demonstrated to lyse primary human monocytes, it has been shown that PSMα peptides and Hld can cause lysis of human monocyte-derived dendritic cells [219]. In addition, assays examining the cytotoxicity of USA300 deletion mutants of Sae that are defective in producing all cytotoxins except PSMs demonstrated these mutants could still cause lysis of primary human monocytes, though significantly less so than wild-type USA300 [63,65]. Collectively, these findings indicate that the majority of cytotoxins produced by *S. aureus* are capable of lysing human monocytes. However, which of these cytotoxins is most important for causing lysis of human monocytes during different contexts of intoxication is not clear.

## 8. Eosinophils and Basophils

Eosinophils and basophils are the least abundant circulating leukocytes, with approximately 50 to 500 eosinophils and 25 to 100 basophils per microliter of adult human blood [119]. These granulocytes are known to play important roles in protection against parasitic infections and in driving allergic responses. In addition, a correlation has been observed between *S. aureus* colonization and atopic diseases that include atopic dermatitis, allergic asthma, and allergic rhinitis [220,221,222,223,224,225,226]. Consequently, studies have examined the influence of *S. aureus* on eosinophil and basophil function with a primary focus on superantigenic proteins produced by this pathogen [227,228,229,230,231,232,233,234,235,236,237,238]. However, the *S. aureus* cytotoxins that target these cell types in humans are almost entirely unknown; a single study has shown that Hla lyses primary human eosinophils [69], while there are no published reports on the susceptibility of human basophils to cytotoxins produced by *S. aureus*. Eosinophils have been shown to express CCR2 [239], CD11b [228,240], CD45 [241], CXCR2 [242], and C5aR [243,244] indicating that these cells are susceptible to HlgAB, HlgCB, LukED, LukGH, and PVL. Basophils are also theoretically susceptible to these bicomponent leukocidins, as they have been shown to express CCR2 [245,246,247], CCR5 [245,246], CD11b [245,248,249,250], CD45 [251], CXCR1 [245,246], CXCR2 [246], and C5aR [243,245,250]. Given that eosinophils and basophils play important roles driving atopic diseases that are associated with *S. aureus* colonization, future studies that define the susceptibility or resistance of these cell types to *S. aureus* cytotoxins may provide insight into the complex manifestation of these diseases.

## 9. Conclusions and Future Studies

Our knowledge of *S. aureus* cytotoxins has made significant advances in the last two decades. These include the characterization of the previously undescribed LukGH, PSMα peptides, and PSMβ peptides, as well as the identification of the host cell receptors recognized by *S. aureus* cytotoxins. However, our understanding of the susceptibility of human peripheral blood cells to each cytotoxin produced by *S. aureus* remains incomplete. Though the cytotoxins that target human neutrophils have been largely defined, the relative susceptibility profiles of other human peripheral blood cell types to these cytotoxins are incomplete or lacking entirely. In addition, how the activation state of host cells influences susceptibility to receptor-dependent cytotoxins is not clear and may be important, as activated host cells alter the expression of cell surface receptors. Though in vitro and ex vivo studies have measured host cell lysis using live *S. aureus*, in vivo stimuli that promote virulence factor expression are absent under these conditions. Moreover, the high genetic variability of *S. aureus* dictates the need for studies comparing cytotoxin production and subsequent host cell lysis caused by different clinical *S. aureus* lineages. Thus, future investigations are needed to fully understand the contribution of each cytotoxin to the lysis of different human peripheral blood cells in vivo by *S. aureus*. Clearly defining the susceptibility profiles of human peripheral blood cells to *S. aureus* cytotoxins will not only advance our understanding of how this bacterium causes a diverse range of human ailments but also lay the foundation for novel therapeutic strategies that limit disease.

## Figures and Tables

**Table 1:** The prevalence and specificity of *S. aureus* cytotoxins against human peripheral blood cell types.

Cytotoxin	Prevalence (%strains)	Cytotoxin Target	Susceptible Human Peripheral Blood Cell Types
α-toxin (Hla)	95% [9,12,55]	ADAM10 [15]	Platelets [56,57,58], Neutrophils [59,60,61], T cells [62,63,64,65,66,67], B cells [62,63,65], Monocytes [62,63,68], and Eosinophils [69]
β-toxin (Hlb)	99% overall [10,70] Deactivated in 87–96% of human isolates [43,45,71]	Receptor independent [7], binds sphingomyelin [28]	Erythrocytes [72], Monocytes [73], proliferating T cells [28]
δ-toxin (Hld)	100% [27]	Receptor independent [14]	Erythrocytes [74]
γ-hemolysin A/B (HlgAB)	99% [13]	CXCR1, CXCR2, CCR2, DARC [16,17]	Erythrocytes [17,54,75,76,77,78], Neutrophils [16,79,80,81,82,83], and Monocytes [16,75]
γ-hemolysin C/B (HlgCB)	99% [13]	C5aR1, C5aR2 [16]	Neutrophils [16,79,80,81,82,83,84], and Monocytes [16,75]
Leukocidin E/D (LukED)	70–80% [13,85]	CCR5, CXCR1, CXCR2, DARC [17,18,19]	Erythrocytes [17,54], Neutrophils [18,79,80,81], T cells [19], and Monocytes [19]
Leukocidin G/H (LukGH or LukAB)	100% [20]	CD11b, HVCN1 [22,23]	Neutrophils [20,21,22,22,23,51,59,79,80,81,86,87,88,89] and Monocytes [23,90]
Panton-Valentine Leukocidin (PVL)	2–3% overall [13] 36% clinical human isolates [91]	C5aR1, C5aR2, CD45 [24,25]	Neutrophils [20,24,25,51,52,79,80,81,82,83,84,86,87,92,93,94,95,96,97,98,99,100] and Monocytes [24,75,83,92,93,101]
Phenol-soluble modulin-α (PSMα) peptides	100% [27]	Receptor independent [14]	Erythrocytes [60,74,102] and Neutrophils [27,59,60,95,103]
Phenol soluble modulin-β (PSMβ) peptides	100% [27]	Receptor independent [14]	Erythrocytes [74]

Legend: DARC: Duffy antigen receptor for chemokines; HVCN1: Voltage-gated hydrogen channel 1.

## Data Availability

The original contributions presented in this study are included in the article. Further inquiries can be directed to the corresponding author.

## References

[1] Kuehnert M.J., Kruszon-Moran D., Hill H.A., McQuillan G., McAllister S.K., Fosheim G., McDougal L.K., Chaitram J., Jensen B., Fridkin S.K. (2006). Prevalence of *Staphylococcus aureus* Nasal Colonization in the United States, 2001–2002. J. Infect. Dis..

[2] Tong S.Y.C., Davis J.S., Eichenberger E., Holland T.L., Fowler V.G. (2015). *Staphylococcus aureus* Infections: Epidemiology, Pathophysiology, Clinical Manifestations, and Management. Clin. Microbiol. Rev..

[3] Clegg J., Soldaini E., McLoughlin R.M., Rittenhouse S., Bagnoli F., Phogat S. (2021). *Staphylococcus aureus* Vaccine Research and Development: The Past, Present and Future, Including Novel Therapeutic Strategies. Front. Immunol..

[4] Miller L.S., Fowler V.G., Shukla S.K., Rose W.E., Proctor R.A. (2020). Development of a Vaccine against *Staphylococcus aureus* Invasive Infections: Evidence Based on Human Immunity, Genetics and Bacterial Evasion Mechanisms. FEMS Microbiol. Rev..

[5] Proctor R.A. (2019). Immunity to *Staphylococcus aureus*: Implications for Vaccine Development. Microbiol. Spectr..

[6] Ikuta K.S., Swetschinski L.R., Aguilar G.R., Sharara F., Mestrovic T., Gray A.P., Weaver N.D., Wool E.E., Han C., Hayoon A.G. (2022). Global Mortality Associated with 33 Bacterial Pathogens in 2019: A Systematic Analysis for the Global Burden of Disease Study 2019. Lancet.

[7] Vandenesch F., Lina G., Henry T. (2012). *Staphylococcus aureus* Hemolysins, Bi-Component Leukocidins, and Cytolytic Peptides: A Redundant Arsenal of Membrane-Damaging Virulence Factors?. Front. Cell. Infect. Microbiol..

[8] Spaan A.N., Van Strijp J.A.G., Torres V.J. (2017). Leukocidins: Staphylococcal Bi-Component Pore-Forming Toxins Find Their Receptors. Nat. Rev. Microbiol..

[9] Ahmad-Mansour N., Loubet P., Pouget C., Dunyach-Remy C., Sotto A., Lavigne J.P., Molle V. (2021). *Staphylococcus aureus* Toxins: An Update on Their Pathogenic Properties and Potential Treatments. Toxins.

[10] Tam K., Torres V.J. (2019). *Staphylococcus aureus* Secreted Toxins and Extracellular Enzymes. Microbiol. Spectr..

[11] Cheung G.Y.C., Bae J.S., Otto M. (2021). Pathogenicity and Virulence of *Staphylococcus aureus*. Virulence.

[12] Oliveira D., Borges A., Simões M. (2018). *Staphylococcus aureus* Toxins and Their Molecular Activity in Infectious Diseases. Toxins.

[13] Alonzo F., Torres V.J. (2014). The Bicomponent Pore-Forming Leucocidins of *Staphylococcus aureus*. Microbiol. Mol. Biol. Rev..

[14] Peschel A., Otto M. (2013). Phenol-Soluble Modulins and Staphylococcal Infection. Nat. Rev. Microbiol..

[15] Wilke G.A., Wardenburg J.B. (2010). Role of a Disintegrin and Metalloprotease 10 in *Staphylococcus aureus* α-Hemolysin–Mediated Cellular Injury. Proc. Natl. Acad. Sci. USA.

[16] Spaan A.N., Vrieling M., Wallet P., Badiou C., Reyes-Robles T., Ohneck E.A., Benito Y., De Haas C.J.C., Day C.J., Jennings M.P. (2014). The Staphylococcal Toxins γ-Haemolysin AB and CB Differentially Target Phagocytes by Employing Specific Chemokine Receptors. Nat. Commun..

[17] Spaan A.N., Reyes-Robles T., Badiou C., Cochet S., Boguslawski K.M., Yoong P., Day C.J., de Haas C.J.C., van Kessel K.P.M., Vandenesch F. (2015). *Staphylococcus aureus* Targets the Duffy Antigen Receptor for Chemokines (DARC) to Lyse Erythrocytes. Cell Host Microbe.

[18] Reyes-Robles T., Alonzo F., Kozhaya L., Lacy D.B., Unutmaz D., Torres V.J. (2013). *Staphylococcus aureus* Leukotoxin ED Targets the Chemokine Receptors CXCR1 and CXCR2 to Kill Leukocytes and Promote Infection. Cell Host Microbe.

[19] Alonzo F., Kozhaya L., Rawlings S.A., Reyes-Robles T., DuMont A.L., Myszka D.G., Landau N.R., Unutmaz D., Torres V.J. (2013). CCR5 Is a Receptor for *Staphylococcus aureus* Leukotoxin ED. Nature.

[20] Ventura C.L., Malachowa N., Hammer C.H., Nardone G.A., Robinson M.A., Kobayashi S.D., DeLeo F.R. (2010). Identification of a Novel *Staphylococcus aureus* Two-Component Leukotoxin Using Cell Surface Proteomics. PLoS ONE.

[21] Dumont A.L., Nygaard T.K., Watkins R.L., Smith A., Kozhaya L., Kreiswirth B.N., Shopsin B., Unutmaz D., Voyich J.M., Torres V.J. (2011). Characterization of a New Cytotoxin That Contributes to *Staphylococcus aureus* Pathogenesis. Mol. Microbiol..

[22] DuMont A.L., Yoong P., Day C.J., Alonzo F., McDonald W.H., Jennings M.P., Torres V.J. (2013). *Staphylococcus aureus* LukAB Cytotoxin Kills Human Neutrophils by Targeting the CD11b Subunit of the Integrin Mac-1. Proc. Natl. Acad. Sci. USA.

[23] Perelman S.S., James D.B.A., Boguslawski K.M., Nelson C.W., Ilmain J.K., Zwack E.E., Prescott R.A., Mohamed A., Tam K., Chan R. (2021). Genetic Variation of Staphylococcal LukAB Toxin Determines Receptor Tropism. Nat. Microbiol..

[24] Spaan A.N., Henry T., van Rooijen W.J.M., Perret M., Badiou C., Aerts P.C., Kemmink J., de Haas C.J.C., van Kessel K.P.M., Vandenesch F. (2013). The Staphylococcal Toxin Panton-Valentine Leukocidin Targets Human C5a Receptors. Cell Host Microbe.

[25] Tromp A.T., Van Gent M., Abrial P., Martin A., Jansen J.P., De Haas C.J.C., Van Kessel K.P.M., Bardoel B.W., Kruse E., Bourdonnay E. (2018). Human CD45 Is an F-Component-Specific Receptor for the Staphylococcal Toxin Panton-Valentine Leukocidin. Nat. Microbiol..

[26] Cheung G.Y.C., Kretschmer D., Queck S.Y., Joo H.-S., Wang R., Duong A.C., Nguyen T.H., Bach T.-H.L., Porter A.R., DeLeo F.R. (2014). Insight into Structure-Function Relationship in Phenol-Soluble Modulins Using an Alanine Screen of the Phenol-Soluble Modulin (PSM) A3 Peptide. FASEB J..

[27] Wang R., Braughton K.R., Kretschmer D., Bach T.-H.L., Queck S.Y., Li M., Kennedy A.D., Dorward D.W., Klebanoff S.J., Peschel A. (2007). Identification of Novel Cytolytic Peptides as Key Virulence Determinants for Community-Associated MRSA. Nat. Med..

[28] Huseby M., Shi K., Brown C.K., Digre J., Mengistu F., Seo K.S., Bohach G.A., Schlievert P.M., Ohlendorf D.H., Earhart C.A. (2007). Structure and Biological Activities of Beta Toxin from *Staphylococcus aureus*. J. Bacteriol..

[29] Rohmer C., Wolz C. (2021). The Role of Hlb-Converting Bacteriophages in *Staphylococcus aureus* Host Adaption. Microb. Physiol..

[30] Spoor L.E., McAdam P.R., Weinert L.A., Rambaut A., Hasman H., Aarestrup F.M., Kearns A.M., Larsen A.R., Skov R.L., Ross Fitzgerald J. (2013). Livestock Origin for a Human Pandemic Clone of Community-Associated Methicillin-Resistant *Staphylococcus aureus*. mBio.

[31] Resch G., François P., Morisset D., Stojanov M., Bonetti E.J., Schrenzel J., Sakwinska O., Moreillon P. (2013). Human-to-Bovine Jump of *Staphylococcus aureus* CC8 Is Associated with the Loss of a β-Hemolysin Converting Prophage and the Acquisition of a New Staphylococcal Cassette Chromosome. PLoS ONE.

[32] Chaguza C., Smith J.T., Bruce S.A., Gibson R., Martin I.W., Andam C.P. (2022). Prophage-Encoded Immune Evasion Factors Are Critical for *Staphylococcus aureus* Host Infection, Switching, and Adaptation. Cell Genomics.

[33] Rohmer C., Dobritz R., Tuncbilek-Dere D., Lehmann E., Gerlach D., George S.E., Bae T., Nieselt K., Wolz C. (2022). Influence of *Staphylococcus aureus* Strain Background on Sa3int Phage Life Cycle Switches. Viruses.

[34] Verkaik N.J., Benard M., Boelens H.A., De Vogel C.P., Nouwen J.L., Verbrugh H.A., Melles D.C., Van Belkum A., Van Wamel W.J.B. (2011). Immune Evasion Cluster-Positive Bacteriophages Are Highly Prevalent among Human *Staphylococcus aureus* Strains, but They Are Not Essential in the First Stages of Nasal Colonization. Clin. Microbiol. Infect..

[35] Matuszewska M., Murray G.G.R., Harrison E.M., Holmes M.A., Weinert L.A. (2020). The Evolutionary Genomics of Host Specificity in *Staphylococcus aureus*. Trends Microbiol..

[36] Bouiller K., Bertrand X., Hocquet D., Chirouze C. (2020). Human Infection of Methicillin-susceptible *Staphylococcus aureus* Cc398: A Review. Microorganisms.

[37] Price L.B., Stegger M., Hasman H., Aziz M., Larsen J., Andersen P.S., Pearson T., Waters A.E., Foster J.T., Schupp J. (2012). *Staphylococcus aureus* CC398: Host Adaptation and Emergence of Methicillin Resistance in Livestock. mBio.

[38] McCarthy A.J., Witney A.A., Gould K.A., Moodley A., Guardabassi L., Voss A., Denis O., Broens E.M., Hinds J., Lindsay J.A. (2011). The Distribution of Mobile Genetic Elements (MGEs) in MRSA CC398 Is Associated with Both Host and Country. Genome Biol. Evol..

[39] Cuny C., Abdelbary M., Layer F., Werner G., Witte W. (2015). Prevalence of the Immune Evasion Gene Cluster in *Staphylococcus aureus* CC398. Vet. Microbiol..

[40] Xia G., Wolz C. (2014). Phages of *Staphylococcus aureus* and Their Impact on Host Evolution. Infect. Genet. Evol..

[41] Howden B.P., Giulieri S.G., Wong Fok Lung T., Baines S.L., Sharkey L.K., Lee J.Y.H., Hachani A., Monk I.R., Stinear T.P. (2023). *Staphylococcus aureus* Host Interactions and Adaptation. Nat. Rev. Microbiol..

[42] Pantůček R., Doškař J., Růžičková V., Kašpárek P., Oráčová E., Kvardová V., Rosypal S. (2004). Identification of Bacteriophage Types and Their Carriage in *Staphylococcus aureus*. Arch. Virol..

[43] Goerke C., Pantucek R., Holtfreter S., Schulte B., Zink M., Grumann D., Bröker B.M., Doskar J., Wolz C. (2009). Diversity of Prophages in Dominant *Staphylococcus aureus* Clonal Lineages. J. Bacteriol..

[44] Goerke C., Wirtz C., Flückiger U., Wolz C. (2006). Extensive Phage Dynamics in *Staphylococcus aureus* Contributes to Adaptation to the Human Host during Infection. Mol. Microbiol..

[45] Van Wamel W.J.B., Rooijakkers S.H.M., Ruyken M., Van Kessel K.P.M., Van Strijp J.A.G. (2006). The Innate Immune Modulators Staphylococcal Complement Inhibitor and Chemotaxis Inhibitory Protein of *Staphylococcus aureus* Are Located on β-Hemolysin-Converting Bacteriophages. J. Bacteriol..

[46] Freer J.H., Arbuthnottt J.P. (1982). Toxins of *Staphylococcus aureus*. Pharmacol. Ther..

[47] Mrochen D.M., Fernandes de Oliveira L.M., Raafat D., Holtfreter S. (2020). *Staphylococcus aureus* Host Tropism and Its Implications for Murine Infection Models. Int. J. Mol. Sci..

[48] Wiseman G.M. (1975). The Hemolysins of *Staphylococcus aureus*. Bacteriol. Rev..

[49] Bernheimer A.W., Avigad L.S., Kim K.S. (1974). Staphylococcal Sphingomyelinase (β-Hemolysin). Ann. N. Y. Acad. Sci..

[50] Elek S.D., Levy E. (1950). Distribution of Hæmolysins in Pathogenic and Non-pathogenic Staphylococci. J. Pathol..

[51] Malachowa N., Kobayashi S.D., Braughton K.R., Whitney A.R., Parnell M.J., Gardner D.J., Deleo F.R. (2012). *Staphylococcus aureus* Leukotoxin GH Promotes Inflammation. J. Infect. Dis..

[52] Löffler B., Hussain M., Grundmeier M., Brück M., Holzinger D., Varga G., Roth J., Kahl B.C., Proctor R.A., Peters G. (2010). *Staphylococcus aureus* Panton-Valentine Leukocidin Is a Very Potent Cytotoxic Factor for Human Neutrophils. PLoS Pathog..

[53] Trstenjak N., Milić D., Graewert M.A., Rouha H., Svergun D., Djinović-Carugo K., Nagy E., Badarau A. (2020). Molecular Mechanism of Leukocidin GH-Integrin CD11b/CD18 Recognition and Species Specificity. Proc. Natl. Acad. Sci. USA.

[54] Adhikari R.P., Kort T., Shulenin S., Kanipakala T., Ganjbaksh N., Roghmann M.-C., Holtsberg F.W., Aman M.J. (2015). Antibodies to *S. Aureus* LukS-PV Attenuated Subunit Vaccine Neutralize a Broad Spectrum of Canonical and Non-Canonical Bicomponent Leukotoxin Pairs. PLoS ONE.

[55] Bennett M.R., Thomsen I.P. (2020). Epidemiological and Clinical Evidence for the Role of Toxins in *S. aureus* Human Disease. Toxins.

[56] Schubert S., Schwertz H., Weyrich A.S., Franks Z.G., Lindemann S., Otto M., Behr H., Loppnow H., Schlitt A., Russ M. (2011). *Staphylococcus aureus* α-Toxin Triggers the Synthesis of B-Cell Lymphoma 3 by Human Platelets. Toxins.

[57] Jahn K., Handtke S., Palankar R., Kohler T.P., Wesche J., Wolff M., Bayer J., Wolz C., Greinacher A., Hammerschmidt S. (2022). α-Hemolysin of *Staphylococcus aureus* Impairs Thrombus Formation. J. Thromb. Haemost..

[58] Sun J., Uchiyama S., Olson J., Morodomi Y., Cornax I., Ando N., Kohno Y., Kyaw M.M.T., Aguilar B., Haste N.M. (2021). Repurposed Drugs Block Toxin-Driven Platelet Clearance by the Hepatic Ashwell-Morell Receptor to Clear *Staphylococcus aureus* Bacteremia. Sci. Transl. Med..

[59] Rungelrath V., Porter A.R., Malachowa N., Freedman B.A., Leung J.M., Voyich J.M., Otto M., Kobayashi S.D., DeLeo F.R. (2021). Further Insight into the Mechanism of Human PMN Lysis Following Phagocytosis of *Staphylococcus aureus*. Microbiol. Spectr..

[60] Li M., Dai Y., Zhu Y., Fu C.-L., Tan V.Y., Wang Y., Wang X., Hong X., Liu Q., Li T. (2016). Virulence Determinants Associated with the Asian Community-Associated Methicillin-Resistant *Staphylococcus aureus* Lineage ST59. Sci. Rep..

[61] Pang Y.Y., Schwartz J., Thoendel M., Ackermann L.W., Horswill A.R., Nauseef W.M. (2010). Agr-Dependent Interactions of *Staphylococcus aureus* USA300 with Human Polymorphonuclear Neutrophils. J. Innate Immun..

[62] Tsuiji M., Shiohara K., Takei Y., Shinohara Y., Nemoto S., Yamaguchi S., Kanto M., Itoh S., Oku T., Miyashita M. (2019). Selective Cytotoxicity of Staphylococcal α-Hemolysin (α-Toxin) against Human Leukocyte Populations. Biol. Pharm. Bull..

[63] Nygaard T.K., Pallister K.B., DuMont A.L., DeWald M., Watkins R.L., Pallister E.Q., Malone C., Griffith S., Horswill A.R., Torres V.J. (2012). Alpha-Toxin Induces Programmed Cell Death of Human T Cells, B Cells, and Monocytes during USA300 Infection. PLoS ONE.

[64] Valeva A., Walev I., Pinkernell M., Walker B., Bayley H., Palmer M., Bhakdi S. (1997). Transmembrane β-Barrel of Staphylococcal α-Toxin Forms in Sensitive but Not in Resistant Cells. Proc. Natl. Acad. Sci. USA.

[65] Kleinhenz M., Li Z., Chidella U., Picard W., Wolfe A., Popelka J., Alexander R., Montgomery C.P. (2024). Toxin-Neutralizing Abs Are Associated with Improved T Cell Function Following Recovery from *Staphylococcus aureus* Infection. JCI Insight.

[66] Jonas D., Walev I., Berger T., Liebetrau M., Palmer M., Bhakdi S. (1994). Novel Path to Apoptosis: Small Transmembrane Pores Created by Staphylococcal Alpha-Toxin in T Lymphocytes Evoke Internucleosomal DNA Degradation. Infect. Immun..

[67] Blümel E., Munir Ahmad S., Nastasi C., Willerslev-Olsen A., Gluud M., Fredholm S., Hu T., Surewaard B.G.J., Lindahl L.M., Fogh H. (2020). *Staphylococcus aureus* Alpha-Toxin Inhibits CD8^+^ T Cell-Mediated Killing of Cancer Cells in Cutaneous T-Cell Lymphoma. Oncoimmunology.

[68] Nygaard T.K., Pallister K.B., Zurek O.W., Voyich J.M. (2013). The Impact of α-Toxin on Host Cell Plasma Membrane Permeability and Cytokine Expression during Human Blood Infection by CA-MRSA USA300. J. Leukoc. Biol..

[69] Prince L.R., Graham K.J., Connolly J., Anwar S., Ridley R., Sabroe I., Foster S.J., Whyte M.K.B. (2012). *Staphylococcus aureus* Induces Eosinophil Cell Death Mediated by α-Hemolysin. PLoS ONE.

[70] Monecke S., Kuhnert P., Hotzel H., Slickers P., Ehricht R. (2007). Microarray Based Study on Virulence-Associated Genes and Resistance Determinants of *Staphylococcus aureus* Isolates from Cattle. Vet. Microbiol..

[71] Aarestrup F.M., Larsen H.D., Eriksen N.H.R., Elsberg C.S., Jensen N.E. (1999). Frequency of α- and β-Haemolysin in *Staphylococcus aureus* of Bovine and Human Origin. Apmis.

[72] Marshall M., Bohach G., Boehm D. (2000). Characterization of *Staphylococcus aureus* Beta-Toxin Induced Leukotoxicity. J. Nat. Toxins.

[73] Walev I., Weller U., Strauch S., Foster T., Bhakdi S. (1996). Selective Killing of Human Monocytes and Cytokine Release Provoked by Sphingomyelinase (Beta-Toxin) of *Staphylococcus aureus*. Infect. Immun..

[74] Cheung G.Y.C., Duong A.C., Otto M. (2012). Direct and Synergistic Hemolysis Caused by Staphylococcus Phenol-Soluble Modulins: Implications for Diagnosis and Pathogenesis. Microbes Infect..

[75] Ferreras M., Höper F., Dalla Serra M., Colin D.A., Prévost G., Menestrina G. (1998). The Interaction of *Staphylococcus aureus* Bi-Component Gamma-Hemolysins and Leucocidins with Cells and Lipid Membranes. Biochim. Biophys. Acta.

[76] Venkatasubramaniam A., Kanipakala T., Ganjbaksh N., Mehr R., Mukherjee I., Krishnan S., Bae T., Aman M.J., Adhikari R.P. (2018). A Critical Role for HlgA in *Staphylococcus aureus* Pathogenesis Revealed by A Switch in the SaeRS Two-Component Regulatory System. Toxins.

[77] Kaneko J., Ozawa T., Tomita T., Kamio Y. (1997). Sequential Binding of Staphylococcal Gamma-Hemolysin to Human Erythrocytes and Complex Formation of the Hemolysin on the Cell Surface. Biosci. Biotechnol. Biochem..

[78] Peng Z., Takeshita M., Shibata N., Tada H., Tanaka Y., Kaneko J. (2018). Rim Domain Loops of Staphylococcal β-Pore Forming Bi-Component Toxin S-Components Recognize Target Human Erythrocytes in a Coordinated Manner. J. Biochem..

[79] Berends E.T.M., Zheng X., Zwack E.E., Ménager M.M., Cammer M., Shopsin B., Torres V.J. (2019). *Staphylococcus aureus* Impairs the Function of and Kills Human Dendritic Cells via the LukAB Toxin. mBio.

[80] Rouha H., Weber S., Janesch P., Maierhofer B., Gross K., Dolezilkova I., Mirkina I., Visram Z.C., Malafa S., Stulik L. (2018). Disarming *Staphylococcus aureus* from Destroying Human Cells by Simultaneously Neutralizing Six Cytotoxins with Two Human Monoclonal Antibodies. Virulence.

[81] Yanai M., Rocha M.A., Matolek A.Z., Chintalacharuvu A., Taira Y., Chintalacharuvu K., Beenhouwer D.O. (2014). Separately or Combined, LukG/LukH Is Functionally Unique Compared to Other Staphylococcal Bicomponent Leukotoxins. PLoS ONE.

[82] Malachowa N., Whitney A.R., Kobayashi S.D., Sturdevant D.E., Kennedy A.D., Braughton K.R., Shabb D.W., Diep B.A., Chambers H.F., Otto M. (2011). Global Changes in *Staphylococcus aureus* Gene Expression in Human Blood. PLoS ONE.

[83] Hodille E., Plesa A., Bourrelly E., Belmont L., Badiou C., Lina G., Dumitrescu O. (2020). Staphylococcal Panton-Valentine Leucocidin and Gamma Haemolysin Target and Lyse Mature Bone Marrow Leucocytes. Toxins.

[84] Spaan A.N., Schiepers A., de Haas C.J.C., van Hooijdonk D.D.J.J., Badiou C., Contamin H., Vandenesch F., Lina G., Gerard N.P., Gerard C. (2015). Differential Interaction of the Staphylococcal Toxins Panton-Valentine Leukocidin and γ-Hemolysin CB with Human C5a Receptors. J. Immunol..

[85] McCarthy A.J., Lindsay J.A. (2013). *Staphylococcus aureus* Innate Immune Evasion Is Lineage-Specific: A Bioinfomatics Study. Infect. Genet. Evol..

[86] Janesch P., Rouha H., Weber S., Malafa S., Gross K., Maierhofer B., Badarau A., Visram Z.C., Stulik L., Nagy E. (2017). Selective Sensitization of Human Neutrophils to LukGH Mediated Cytotoxicity by *Staphylococcus aureus* and IL-8. J. Infect..

[87] DuMont A.L., Yoong P., Surewaard B.G.J., Benson M.A., Nijland R., van Strijp J.A.G., Torres V.J. (2013). *Staphylococcus aureus* Elaborates Leukocidin AB to Mediate Escape from within Human Neutrophils. Infect. Immun..

[88] Yang D., Ho Y.X., Cowell L.M., Jilani I., Foster S.J., Prince L.R. (2019). A Genome-Wide Screen Identifies Factors Involved in *S. aureus*-Induced Human Neutrophil Cell Death and Pathogenesis. Front. Immunol..

[89] Nygaard T.K., Borgogna T.R., Pallister K.B., Predtechenskaya M., Burroughs O.S., Gao A., Lubick E.G., Voyich J.M. (2024). The Relative Importance of Cytotoxins Produced by Methicillin-Resistant *Staphylococcus aureus* Strain USA300 for Causing Human PMN Destruction. Microorganisms.

[90] Melehani J.H., James D.B.A., DuMont A.L., Torres V.J., Duncan J.A. (2015). *Staphylococcus aureus* Leukocidin A/B (LukAB) Kills Human Monocytes via Host NLRP3 and ASC When Extracellular, but Not Intracellular. PLoS Pathog..

[91] Brown M.L., O’Hara F.P., Close N.M., Mera R.M., Miller L.A., Suaya J.A., Amrine-Madsen H. (2012). Prevalence and Sequence Variation of Panton-Valentine Leukocidin in Methicillin-Resistant and Methicillin-Susceptible *Staphylococcus aureus* Strains in the United States. J. Clin. Microbiol..

[92] Grebe T., Sarkari M.T., Cherkaoui A., Schaumburg F. (2024). Exploration of Compounds to Inhibit the Panton-Valentine Leukocidin of *Staphylococcus aureus*. Med. Microbiol. Immunol..

[93] Holzinger D., Gieldon L., Mysore V., Nippe N., Taxman D.J., Duncan J.A., Broglie P.M., Marketon K., Austermann J., Vogl T. (2012). *Staphylococcus aureus* Panton-Valentine Leukocidin Induces an Inflammatory Response in Human Phagocytes via the NLRP3 Inflammasome. J. Leukoc. Biol..

[94] Graves S.F., Kobayashi S.D., Braughton K.R., Diep B.A., Chambers H.F., Otto M., Deleo F.R. (2010). Relative Contribution of Panton-Valentine Leukocidin to PMN Plasma Membrane Permeability and Lysis Caused by USA300 and USA400 Culture Supernatants. Microbes Infect..

[95] Hongo I., Baba T., Oishi K., Morimoto Y., Ito T., Hiramatsu K. (2009). Phenol-Soluble Modulin Alpha 3 Enhances the Human Neutrophil Lysis Mediated by Panton-Valentine Leukocidin. J. Infect. Dis..

[96] Genestier A.-L., Michallet M.-C., Prévost G., Bellot G., Chalabreysse L., Peyrol S., Thivolet F., Etienne J., Lina G., Vallette F.M. (2005). *Staphylococcus aureus* Panton-Valentine Leukocidin Directly Targets Mitochondria and Induces Bax-Independent Apoptosis of Human Neutrophils. J. Clin. Investig..

[97] Mairpady Shambat S., Chen P., Nguyen Hoang A.T., Bergsten H., Vandenesch F., Siemens N., Lina G., Monk I.R., Foster T.J., Arakere G. (2015). Modelling Staphylococcal Pneumonia in a Human 3D Lung Tissue Model System Delineates Toxin-Mediated Pathology. Dis. Models Mech..

[98] Gauduchon V., Cozon G., Vandenesch F., Genestier A., Eyssade N., Peyrol S., Etienne J., Lina G. (2004). Neutralization of *Staphylococcus aureus* Panton Valentine Leukocidin by Intravenous Immunoglobulin In Vitro. J. Infect. Dis..

[99] Hermos C.R., Yoong P., Pier G.B. (2010). High Levels of Antibody to Panton-Valentine Leukocidin Are Not Associated with Resistance to *Staphylococcus aureus*—Associated Skin and Soft-Tissue Infection. Clin. Infect. Dis..

[100] Meyer F., Girardot R., Piémont Y., Prévost G., Colin D.A. (2009). Analysis of the Specificity of Panton-Valentine Leucocidin and Gamma-Hemolysin F Component Binding. Infect. Immun..

[101] Jeannoel M., Casalegno J.-S., Ottmann M., Badiou C., Dumitrescu O., Lina B., Lina G. (2018). Synergistic Effects of Influenza and *Staphylococcus aureus* Toxins on Inflammation Activation and Cytotoxicity in Human Monocytic Cell Lines. Toxins.

[102] Wu Y., Chen T., Wang Y., Huang M., Wang Y., Luo Z. (2023). New Insight into the Virulence and Inflammatory Response of *Staphylococcus aureus* Strains Isolated from Diabetic Foot Ulcers. Front. Cell. Infect. Microbiol..

[103] Surewaard B.G.J., de Haas C.J.C., Vervoort F., Rigby K.M., DeLeo F.R., Otto M., van Strijp J.A.G., Nijland R. (2013). Staphylococcal Alpha-Phenol Soluble Modulins Contribute to Neutrophil Lysis after Phagocytosis. Cell. Microbiol..

[104] Haag A.F., Bagnoli F. (2017). The Role of Two-Component Signal Transduction Systems in *Staphylococcus aureus* Virulence Regulation. Curr. Top. Microbiol. Immunol..

[105] Bleul L., Francois P., Wolz C. (2021). Two-Component Systems of S. Aureus: Signaling and Sensing Mechanisms. Genes.

[106] Kuroda M., Ohta T., Uchiyama I., Baba T., Yuzawa H., Kobayashi I., Cui L., Oguchi A., Aoki K., Nagai Y. (2001). Whole Genome Sequencing of Meticillin-Resistant *Staphylococcus aureus*. Lancet.

[107] Le K.Y., Otto M. (2015). Quorum-Sensing Regulation in Staphylococci—An Overview. Front. Microbiol..

[108] Yarwood J.M., Schlievert P.M. (2003). Quorum Sensing in Staphylococcus Infections. J. Clin. Investig..

[109] Novick R.P., Ross H.F., Projan S.J., Kornblum J., Kreiswirth B., Moghazeh S. (1993). Synthesis of Staphylococcal Virulence Factors Is Controlled by a Regulatory RNA Molecule. EMBO J..

[110] Boisset S., Geissmann T., Huntzinger E., Fechter P., Bendridi N., Possedko M., Chevalier C., Helfer A.C., Benito Y., Jacquier A. (2007). *Staphylococcus aureus* RNAIII Coordinately Represses the Synthesis of Virulence Factors and the Transcription Regulator Rot by an Antisense Mechanism. Genes Dev..

[111] Queck S.Y., Jameson-Lee M., Villaruz A.E., Bach T.-H.L., Khan B.A., Sturdevant D.E., Ricklefs S.M., Li M., Otto M. (2008). RNAIII-Independent Target Gene Control by the Agr Quorum-Sensing System: Insight into the Evolution of Virulence Regulation in *Staphylococcus aureus*. Mol. Cell.

[112] Flack C.E., Zurek O.W., Meishery D.D., Pallister K.B., Malone C.L., Horswill A.R., Voyich J.M. (2014). Differential Regulation of Staphylococcal Virulence by the Sensor Kinase SaeS in Response to Neutrophil-Derived Stimuli. Proc. Natl. Acad. Sci. USA.

[113] Nygaard T.K., Borgogna T.R., Sward E.W., Guerra F.E., Dankoff J.G., Collins M.M., Pallister K.B., Chen L., Kreiswirth B.N., Voyich J.M. (2018). Aspartic Acid Residue 51 of SaeR Is Essential for *Staphylococcus aureus* Virulence. Front. Microbiol..

[114] Nygaard T.K., Pallister K.B., Ruzevich P., Griffith S., Vuong C., Voyich J.M. (2010). SaeR Binds a Consensus Sequence within Virulence Gene Promoters to Advance USA300 Pathogenesis. J. Infect. Dis..

[115] Voyich J.M., Vuong C., DeWald M., Nygaard T.K., Kocianova S., Griffith S., Jones J., Iverson C., Sturdevant D.E., Braughton K.R. (2009). The SaeR/S Gene Regulatory System Is Essential for Innate Immune Evasion by *Staphylococcus aureus*. J. Infect. Dis..

[116] Liu Q., Yeo W.-S., Bae T. (2016). The SaeRS Two-Component System of *Staphylococcus aureus*. Genes.

[117] Geiger T., Goerke C., Mainiero M., Kraus D., Wolz C. (2008). The Virulence Regulator Sae of *Staphylococcus aureus*: Promoter Activities and Response to Phagocytosis-Related Signals. J. Bacteriol..

[118] Boguslawski K.M., McKeown A.N., Day C.J., Lacey K.A., Tam K., Vozhilla N., Kim S.Y., Jennings M.P., Koralov S.B., Elde N.C. (2020). Exploiting Species Specificity to Understand the Tropism of a Human-Specific Toxin. Sci. Adv..

[119] Pagana K.D., Pagana T.J., Pagana T.N. (2024). Mosby’s^®^ Diagnostic and Laboratory Test Reference.

[120] Skaar E.P., Schneewind O. (2004). Iron-Regulated Surface Determinants (Isd) of *Staphylococcus aureus*: Stealing Iron from Heme. Microbes Infect..

[121] Van Dijk M.C., De Kruijff R.M., Hagedoorn P.-L. (2022). The Role of Iron in *Staphylococcus aureus* Infection and Human Disease: A Metal Tug of War at the Host—Microbe Interface. Front. Cell Dev. Biol..

[122] Torres V.J., Pishchany G., Humayun M., Schneewind O., Skaar E.P. (2006). *Staphylococcus aureus* IsdB Is a Hemoglobin Receptor Required for Heme Iron Utilization. J. Bacteriol..

[123] Skaar E.P., Humayun M., Bae T., DeBord K.L., Schneewind O. (2004). Iron-Source Preference of *Staphylococcus aureus* Infections. Science.

[124] Hildebrand A., Pohl M., Bhakdi S. (1991). *Staphylococcus aureus* Alpha-Toxin. Dual Mechanism of Binding to Target Cells. J. Biol. Chem..

[125] Sanger R., Race R.R., Jack J. (1955). The Duffy Blood Groups of New York Negroes: The Phenotype Fy (A−b−). Br. J. Haematol..

[126] Miller L.H., Mason S.J., Clyde D.F., McGinniss M.H. (1976). The Resistance Factor to Plasmodium Vivax in Blacks. N. Engl. J. Med..

[127] Wolfmeier H., Mansour S.C., Liu L.T., Pletzer D., Draeger A., Babiychuk E.B., Hancock R.E.W. (2018). Liposomal Therapy Attenuates Dermonecrosis Induced by Community-Associated Methicillin-Resistant *Staphylococcus aureus* by Targeting α-Type Phenol-Soluble Modulins and α-Hemolysin. EBioMedicine.

[128] Hébert G.A., Hancock G.A. (1985). Synergistic Hemolysis Exhibited by Species of Staphylococci. J. Clin. Microbiol..

[129] Mainiero M., Goerke C., Geiger T., Gonser C., Herbert S., Wolz C. (2010). Differential Target Gene Activation by the *Staphylococcus aureus* Two-Component System saeRS. J. Bacteriol..

[130] Guo L., Rondina M.T. (2019). The Era of Thromboinflammation: Platelets Are Dynamic Sensors and Effector Cells During Infectious Diseases. Front. Immunol..

[131] Li C., Li J., Ni H. (2020). Crosstalk Between Platelets and Microbial Pathogens. Front. Immunol..

[132] Yeaman M.R. (2014). Platelets: At the Nexus of Antimicrobial Defence. Nat. Rev. Microbiol..

[133] Hamzeh-Cognasse H., Damien P., Chabert A., Pozzetto B., Cognasse F., Garraud O. (2015). Platelets and Infections—Complex Interactions with Bacteria. Front. Immunol..

[134] van der Meijden P.E.J., Heemskerk J.W.M. (2019). Platelet Biology and Functions: New Concepts and Clinical Perspectives. Nat. Rev. Cardiol..

[135] Douglas-Louis R., Lou M., Lee B., Minejima E., Bubeck-Wardenburg J., Wong-Beringer A. (2023). Prognostic Significance of Early Platelet Dynamics in *Staphylococcus aureus* Bacteremia. BMC Infect. Dis..

[136] Gafter-Gvili A., Mansur N., Bivas A., Zemer-Wassercug N., Bishara J., Leibovici L., Paul M. (2011). Thrombocytopenia in *Staphylococcus aureus* Bacteremia: Risk Factors and Prognostic Importance. Mayo Clin. Proc..

[137] Gaertner F., Ahmad Z., Rosenberger G., Fan S., Nicolai L., Busch B., Yavuz G., Luckner M., Ishikawa-Ankerhold H., Hennel R. (2017). Migrating Platelets Are Mechano-Scavengers That Collect and Bundle Bacteria. Cell.

[138] Kraemer B.F., Campbell R.A., Schwertz H., Cody M.J., Franks Z., Tolley N.D., Kahr W.H.A., Lindemann S., Seizer P., Yost C.C. (2011). Novel Anti-Bacterial Activities of β-Defensin 1 in Human Platelets: Suppression of Pathogen Growth and Signaling of Neutrophil Extracellular Trap Formation. PLoS Pathog..

[139] Fitzgerald J.R., Loughman A., Keane F., Brennan M., Knobel M., Higgins J., Visai L., Speziale P., Cox D., Foster T.J. (2006). Fibronectin-Binding Proteins of *Staphylococcus aureus* Mediate Activation of Human Platelets via Fibrinogen and Fibronectin Bridges to Integrin GPIIb/IIIa and IgG Binding to the FcγRIIa Receptor. Mol. Microbiol..

[140] Vanassche T., Kauskot A., Verhaegen J., Peetermans W.E., van Ryn J., Schneewind O., Hoylaerts M.F., Verhamme P. (2012). Fibrin Formation by Staphylothrombin Facilitates *Staphylococcus aureus*-Induced Platelet Aggregation. Thromb. Haemost..

[141] Miajlovic H., Loughman A., Brennan M., Cox D., Foster T.J. (2007). Both Complement- and Fibrinogen-Dependent Mechanisms Contribute to Platelet Aggregation Mediated by *Staphylococcus aureus* Clumping Factor B. Infect. Immun..

[142] O’Brien L., Kerrigan S.W., Kaw G., Hogan M., Penadés J., Litt D., Fitzgerald D.J., Foster T.J., Cox D. (2002). Multiple Mechanisms for the Activation of Human Platelet Aggregation by *Staphylococcus aureus*: Roles for the Clumping Factors ClfA and ClfB, the Serine-Aspartate Repeat Protein SdrE and Protein A. Mol. Microbiol..

[143] Hawiger J., Steckley S., Hammond D., Cheng C., Timmons S., Glick A.D., Des Prez R.M. (1979). Staphylococci-Induced Human Platelet Injury Mediated by Protein A and Immunoglobulin G Fc Fragment Receptor. J. Clin. Investig..

[144] De Haas C.J.C., Weeterings C., Vughs M.M., De Groot P.G., Van Strijp J.A., Lisman T. (2009). Staphylococcal Superantigen-like 5 Activates Platelets and Supports Platelet Adhesion under Flow Conditions, Which Involves Glycoprotein Ibα and αIIbβ3. J. Thromb. Haemost..

[145] Surewaard B.G.J., Thanabalasuriar A., Zeng Z., Tkaczyk C., Cohen T.S., Bardoel B.W., Jorch S.K., Deppermann C., Bubeck Wardenburg J., Davis R.P. (2018). α-Toxin Induces Platelet Aggregation and Liver Injury during *Staphylococcus aureus* Sepsis. Cell Host Microbe.

[146] Liesenborghs L., Verhamme P., Vanassche T. (2018). *Staphylococcus aureus*, Master Manipulator of the Human Hemostatic System. J. Thromb. Haemost..

[147] Colciaghi F., Borroni B., Pastorino L., Marcello E., Zimmermann M., Cattabeni F., Padovani A., Di Luca M. (2002). α-Secretase ADAM10 as Well as αAPPs Is Reduced in Platelets and CSF of Alzheimer Disease Patients. Mol. Med..

[148] Raab S., Kropp K.N., Steinle A., Klein G., Kanz L., Kopp H.-G., Salih H.R. (2014). Platelet-Derived Proteases ADAM10 and ADAM17 Impair NK Cell Immunosurveillance of Metastasizing Tumor Cells by Diminishing NKG2D Ligand Surface Expression. Blood.

[149] Philippeaux M.M., Vesin C., Tacchini-Cottier F., Piguet P.F. (1996). Activated Human Platelets Express Beta2 Integrin. Eur. J. Haematol..

[150] Nording H., Baron L., Haberthür D., Emschermann F., Mezger M., Sauter M., Sauter R., Patzelt J., Knoepp K., Nording A. (2021). The C5a/C5a Receptor 1 Axis Controls Tissue Neovascularization through CXCL4 Release from Platelets. Nat. Commun..

[151] Patzelt J., Mueller K.A.L., Breuning S., Karathanos A., Schleicher R., Seizer P., Gawaz M., Langer H.F., Geisler T. (2015). Expression of Anaphylatoxin Receptors on Platelets in Patients with Coronary Heart Disease. Atherosclerosis.

[152] Apostolidis S.A., Sarkar A., Giannini H.M., Goel R.R., Mathew D., Suzuki A., Baxter A.E., Greenplate A.R., Alanio C., Abdel-Hakeem M. (2021). Signaling through FcγRIIA and the C5a-C5aR Pathway Mediates Platelet Hyperactivation in COVID-19. bioRxiv.

[153] Guerra F.E., Borgogna T.R., Patel D.M., Sward E.W., Voyich J.M. (2017). Epic Immune Battles of History: Neutrophils vs. Staphylococcus aureus. Front. Cell. Infect. Microbiol..

[154] Nygaard T., Malachowa N., Kobayashi S.D., DeLeo F.R., Segal B.H. (2018). Phagocytes. Management of Infections in the Immunocompromised Host.

[155] Karavolos M.H., Horsburgh M.J., Ingham E., Foster S.J. (2003). Role and Regulation of the Superoxide Dismutases of *Staphylococcus aureus*. Microbiology.

[156] Horsburgh M.J., Clements M.O., Crossley H., Ingham E., Foster S.J. (2001). PerR Controls Oxidative Stress Resistance and Iron Storage Proteins and Is Required for Virulence in *Staphylococcus aureus*. Infect. Immun..

[157] de Jong N.W.M., Ramyar K.X., Guerra F.E., Nijland R., Fevre C., Voyich J.M., McCarthy A.J., Garcia B.L., van Kessel K.P.M., van Strijp J.A.G. (2017). Immune Evasion by a Staphylococcal Inhibitor of Myeloperoxidase. Proc. Natl. Acad. Sci. USA.

[158] Guerra F.E., Addison C.B., de Jong N.W.M., Azzolino J., Pallister K.B., van Strijp J.A.G., Voyich J.M. (2016). *Staphylococcus aureus* SaeR/S-Regulated Factors Reduce Human Neutrophil Reactive Oxygen Species Production. J. Leukoc. Biol..

[159] Voyich J.M., Braughton K.R., Sturdevant D.E., Whitney A.R., Saïd-Salim B., Porcella S.F., Long R.D., Dorward D.W., Gardner D.J., Kreiswirth B.N. (2005). Insights into Mechanisms Used by *Staphylococcus aureus* to Avoid Destruction by Human Neutrophils. J. Immunol..

[160] Kobayashi S.D., Braughton K.R., Palazzolo-Ballance A.M., Kennedy A.D., Sampaio E., Kristosturyan E., Whitney A.R., Sturdevant D.E., Dorward D.W., Holland S.M. (2010). Rapid Neutrophil Destruction Following Phagocytosis of *Staphylococcus aureus*. J. Innate Immun..

[161] Seifert A., Düsterhöft S., Wozniak J., Koo C.Z., Tomlinson M.G., Nuti E., Rossello A., Cuffaro D., Yildiz D., Ludwig A. (2020). The Metalloproteinase ADAM10 Requires Its Activity to Sustain Surface Expression. Cell. Mol. Life Sci..

[162] Metzemaekers M., Gouwy M., Proost P. (2020). Neutrophil Chemoattractant Receptors in Health and Disease: Double-Edged Swords. Cell. Mol. Immunol..

[163] Futosi K., Fodor S., Mócsai A. (2013). Neutrophil Cell Surface Receptors and Their Intracellular Signal Transduction Pathways. Int. Immunopharmacol..

[164] Nygaard T.K., DeLeo F.R., Voyich J.M. (2008). Community-Associated Methicillin-Resistant *Staphylococcus aureus* Skin Infections: Advances toward Identifying the Key Virulence Factors. Curr. Opin. Infect. Dis..

[165] Zheng X., Marsman G., Lacey K.A., Chapman J.R., Goosmann C., Ueberheide B.M., Torres V.J. (2021). The Cell Envelope of *Staphylococcus aureus* Selectively Controls the Sorting of Virulence Factors. Nat. Commun..

[166] DuMont A.L., Yoong P., Liu X., Day C.J., Chumbler N.M., James D.B.A., Alonzo F., Bode N.J., Lacy D.B., Jennings M.P. (2014). Identification of a Crucial Residue Required for *Staphylococcus aureus* LukAB Cytotoxicity and Receptor Recognition. Infect. Immun..

[167] Badarau A., Rouha H., Malafa S., Logan D.T., Håkansson M., Stulik L., Dolezilkova I., Teubenbacher A., Gross K., Maierhofer B. (2015). Structure-Function Analysis of Heterodimer Formation, Oligomerization, and Receptor Binding of the *Staphylococcus aureus* Bi-Component Toxin LukGH. J. Biol. Chem..

[168] Fitzgerald J.R., Sturdevant D.E., Mackie S.M., Gill S.R., Musser J.M. (2001). Evolutionary Genomics of *Staphylococcus aureus*: Insights into the Origin of Methicillin-Resistant Strains and the Toxic Shock Syndrome Epidemic. Proc. Natl. Acad. Sci. USA.

[169] Pivard M., Caldelari I., Brun V., Croisier D., Jaquinod M., Anzala N., Gilquin B., Teixeira C., Benito Y., Couzon F. (2023). Complex Regulation of Gamma-Hemolysin Expression Impacts *Staphylococcus aureus* Virulence. Microbiol. Spectr..

[170] Dastgheyb S.S., Otto M. (2015). Staphylococcal Adaptation to Diverse Physiologic Niches: An Overview of Transcriptomic and Phenotypic Changes in Different Biological Environments. Future Microbiol..

[171] Den Reijer P.M., Lemmens-den Toom N., Kant S., Snijders S.V., Boelens H., Tavakol M., Verkaik N.J., Van Belkum A., Verbrugh H.A., Van Wamel W.J.B. (2013). Characterization of the Humoral Immune Response during *Staphylococcus aureus* Bacteremia and Global Gene Expression by *Staphylococcus aureus* in Human Blood. PLoS ONE.

[172] Loughman J.A., Fritz S.A., Storch G.A., Hunstad D.A. (2009). Virulence Gene Expression in Human Community-Acquired *Staphylococcus aureus* Infection. J. Infect. Dis..

[173] Date S.V., Modrusan Z., Lawrence M., Morisaki J.H., Toy K., Shah I.M., Kim J., Park S., Xu M., Basuino L. (2014). Global Gene Expression of Methicillin-Resistant *Staphylococcus aureus* USA300 during Human and Mouse Infection. J. Infect. Dis..

[174] Dong C. (2021). Cytokine Regulation and Function in T Cells. Annu. Rev. Immunol..

[175] Armentrout E.I., Liu G.Y., Martins G.A. (2020). T Cell Immunity and the Quest for Protective Vaccines against *Staphylococcus aureus* Infection. Microorganisms.

[176] Kolata J.B., Kühbandner I., Link C., Normann N., Vu C.H., Steil L., Weidenmaier C., Bröker B.M. (2015). The Fall of a Dogma? Unexpected High T-Cell Memory Response to *Staphylococcus aureus* in Humans. J. Infect. Dis..

[177] Miller L.S., Cho J.S. (2011). Immunity against *Staphylococcus aureus* Cutaneous Infections. Nat. Rev. Immunol..

[178] Hendriks A., Mnich M.E., Clemente B., Cruz A.R., Tavarini S., Bagnoli F., Soldaini E. (2021). *Staphylococcus aureus*-Specific Tissue-Resident Memory CD4^+^ T Cells Are Abundant in Healthy Human Skin. Front. Immunol..

[179] Marrack P., Kappler J. (1990). The Staphylococcal Enterotoxins and Their Relatives. Science.

[180] Proft T., Fraser J.D. (2003). Bacterial Superantigens. Clin. Exp. Immunol..

[181] Deacy A.M., Gan S.K.-E., Derrick J.P. (2021). Superantigen Recognition and Interactions: Functions, Mechanisms and Applications. Front. Immunol..

[182] Choi Y., Lafferty J.A., Clements J.R., Todd J.K., Gelfand E.W., Kappler J., Marrack P., Kotzin B.L. (1990). Selective Expansion of T Cells Expressing V Beta 2 in Toxic Shock Syndrome. J. Exp. Med..

[183] Kotzin B.L., Leung D.Y., Kappler J., Marrack P. (1993). Superantigens and Their Potential Role in Human Disease. Adv. Immunol..

[184] Sezin T., Selvakumar B., Scheffold A. (2022). The Role of A Disintegrin and Metalloproteinase (ADAM)-10 in T Helper Cell Biology. Biochim. Biophys. Acta (BBA)-Mol. Cell Res..

[185] Bonecchi R., Bianchi G., Bordignon P.P., D’Ambrosio D., Lang R., Borsatti A., Sozzani S., Allavena P., Gray P.A., Mantovani A. (1998). Differential Expression of Chemokine Receptors and Chemotactic Responsiveness of Type 1 T Helper Cells (Th1s) and Th2s. J. Exp. Med..

[186] Frade J.M., Mellado M., del Real G., Gutierrez-Ramos J.C., Lind P., Martinez-A C. (1997). Characterization of the CCR2 Chemokine Receptor: Functional CCR2 Receptor Expression in B Cells. J. Immunol..

[187] Nansen A., Marker O., Bartholdy C., Thomsen A.R. (2000). CCR2^+^ and CCR5^+^ CD8^+^ T Cells Increase during Viral Infection and Migrate to Sites of Infection. Eur. J. Immunol..

[188] Fiorentini S., Licenziati S., Alessandri G., Castelli F., Caligaris S., Bonafede M., Grassi M., Garrafa E., Balsari A., Turano A. (2001). CD11b Expression Identifies CD8^+^CD28^+^ T Lymphocytes with Phenotype and Function of Both Naive/Memory and Effector Cells. J. Immunol..

[189] Lin Y., Roberts T.J., Sriram V., Cho S., Brutkiewicz R.R. (2003). Myeloid Marker Expression on Antiviral CD8^+^ T Cells Following an Acute Virus Infection. Eur. J. Immunol..

[190] Hermiston M.L., Xu Z., Weiss A. (2003). CD45: A Critical Regulator of Signaling Thresholds in Immune Cells. Annu. Rev. Immunol..

[191] Francis J.N., Jacobson M.R., Lloyd C.M., Sabroe I., Durham S.R., Till S.J. (2004). CXCR1^+^CD4^+^ T Cells in Human Allergic Disease. J. Immunol..

[192] Takata H., Tomiyama H., Fujiwara M., Kobayashi N., Takiguchi M. (2004). Cutting Edge: Expression of Chemokine Receptor CXCR1 on Human Effector CD8^+^ T Cells1. J. Immunol..

[193] Gasser O., Missiou A., Eken C., Hess C. (2005). Human CD8^+^ T Cells Store CXCR1 in a Distinct Intracellular Compartment and Up-Regulate It Rapidly to the Cell Surface upon Activation. Blood.

[194] Nataf S., Davoust N., Ames R.S., Barnum S.R. (1999). Human T Cells Express the C5a Receptor and Are Chemoattracted to C5a1. J. Immunol..

[195] Verghese D.A., Chun N., Paz K., Fribourg M., Woodruff T.M., Flynn R., Hu Y., Xiong H., Zhang W., Yi Z. (2018). C5aR1 Regulates T Follicular Helper Differentiation and Chronic Graft-versus-Host Disease Bronchiolitis Obliterans. JCI Insight.

[196] Pozzi C., Lofano G., Mancini F., Soldaini E., Speziale P., De Gregorio E., Rappuoli R., Bertholet S., Grandi G., Bagnoli F. (2015). Phagocyte Subsets and Lymphocyte Clonal Deletion behind Ineffective Immune Response to *Staphylococcus aureus*. FEMS Microbiol. Rev..

[197] Karauzum H., Datta S.K. (2017). Adaptive Immunity against *Staphylococcus aureus*. Curr. Top. Microbiol. Immunol..

[198] Spellberg B., Daum R. (2012). Development of a Vaccine against *Staphylococcus aureus*. Semin. Immunopathol..

[199] Deisenhofer J. (1981). Crystallographic Refinement and Atomic Models of a Human Fc Fragment and Its Complex with Fragment B of Protein A from *Staphylococcus aureus* at 2.9- and 2.8-.ANG. Resolution. Biochemistry.

[200] Moks T., Abrahmsén L., Nilsson B., Hellman U., Sjöquist J., Uhlén M. (1986). Staphylococcal Protein A Consists of Five IgG-Binding Domains. Eur. J. Biochem..

[201] Zhang L., Jacobsson K., Vasi J., Lindberg M., Frykberg L. (1998). A Second IgG-Binding Protein in *Staphylococcus aureus*. Microbiology.

[202] Atkins K.L., Burman J.D., Chamberlain E.S., Cooper J.E., Poutrel B., Bagby S., Jenkins A.T.A., Feil E.J., van den Elsen J.M.H.S. (2008). Aureus IgG-Binding Proteins SpA and Sbi: Host Specificity and Mechanisms of Immune Complex Formation. Mol. Immunol..

[203] Romagnani S., Giudizi M.G., del Prete G., Maggi E., Biagiotti R., Almerigogna F., Ricci M. (1982). Demonstration on Protein A of Two Distinct Immunoglobulin-Binding Sites and Their Role in the Mitogenic Activity of *Staphylococcus aureus* Cowan I on Human B Cells. J. Immunol..

[204] Silverman G.J., Goodyear C.S. (2002). A Model B-Cell Superantigen and the Immunobiology of B Lymphocytes. Clin. Immunol..

[205] Goodyear C.S., Silverman G.J. (2003). Death by a B Cell Superantigen. J. Exp. Med..

[206] Goodyear C.S., Silverman G.J. (2004). Staphylococcal Toxin Induced Preferential and Prolonged In Vivo Deletion of Innate-like B Lymphocytes. Proc. Natl. Acad. Sci. USA.

[207] Lownik J.C., Luker A.J., Damle S.R., Cooley L.F., El Sayed R., Hutloff A., Pitzalis C., Martin R.K., El Shikh M.E.M., Conrad D.H. (2017). ADAM10-Mediated ICOSL Shedding on B Cells Is Necessary for Proper T Cell ICOS Regulation and TFH Responses. J. Immunol..

[208] Jakubzick C.V., Randolph G.J., Henson P.M. (2017). Monocyte Differentiation and Antigen-Presenting Functions. Nat. Rev. Immunol..

[209] Ardura M.I., Banchereau R., Mejias A., Di Pucchio T., Glaser C., Allantaz F., Pascual V., Banchereau J., Chaussabel D., Ramilo O. (2009). Enhanced Monocyte Response and Decreased Central Memory T Cells in Children with Invasive *Staphylococcus aureus* Infections. PLoS ONE.

[210] Zwack E.E., Chen Z., Devlin J.C., Li Z., Zheng X., Weinstock A., Lacey K.A., Fisher E.A., Fenyö D., Ruggles K.V. (2022). *Staphylococcus aureus* Induces a Muted Host Response in Human Blood That Blunts the Recruitment of Neutrophils. Proc. Natl. Acad. Sci. USA.

[211] Kubica M., Guzik K., Koziel J., Zarebski M., Richter W., Gajkowska B., Golda A., Maciag-Gudowska A., Brix K., Shaw L. (2008). A Potential New Pathway for *Staphylococcus aureus* Dissemination: The Silent Survival of *S. aureus* Phagocytosed by Human Monocyte-Derived Macrophages. PLoS ONE.

[212] Musilova J., Mulcahy M.E., Kuijk M.M., McLoughlin R.M., Bowie A.G. (2019). Toll-like Receptor 2–Dependent Endosomal Signaling by *Staphylococcus aureus* in Monocytes Induces Type I Interferon and Promotes Intracellular Survival. J. Biol. Chem..

[213] Thwaites G.E., Gant V. (2011). Are Bloodstream Leukocytes Trojan Horses for the Metastasis of *Staphylococcus aureus*?. Nat. Rev. Microbiol..

[214] Wong K.L., Tai J.J.-Y., Wong W.-C., Han H., Sem X., Yeap W.-H., Kourilsky P., Wong S.-C. (2011). Gene Expression Profiling Reveals the Defining Features of the Classical, Intermediate, and Nonclassical Human Monocyte Subsets. Blood.

[215] Schaeffer V., Cuschieri J., Garcia I., Knoll M., Billgren J., Jelacic S., Bulger E., Maier R. (2007). The priming effect of C5a on monocytes is predominantly mediated by the p38 MAPK pathway. Shock.

[216] Möller-Hackbarth K., Dewitz C., Schweigert O., Trad A., Garbers C., Rose-John S., Scheller J. (2013). A Disintegrin and Metalloprotease (ADAM) 10 and ADAM17 Are Major Sheddases of T Cell Immunoglobulin and Mucin Domain 3 (Tim-3). J. Biol. Chem..

[217] Trebst C., Sørensen T.L., Kivisäkk P., Cathcart M.K., Hesselgesser J., Horuk R., Sellebjerg F., Lassmann H., Ransohoff R.M. (2001). CCR1^+^/CCR5^+^ Mononuclear Phagocytes Accumulate in the Central Nervous System of Patients with Multiple Sclerosis. Am. J. Pathol..

[218] Ales E., Sackstein R. (2022). Analysis of CD45 Isoforms Displayed By Human Peripheral Blood Mononuclear Cells. Blood.

[219] Richardson J.R., Armbruster N.S., Günter M., Henes J., Autenrieth S.E. (2018). *Staphylococcus aureus* PSM Peptides Modulate Human Monocyte-Derived Dendritic Cells to Prime Regulatory T Cells. Front. Immunol..

[220] Vickery T.W., Ramakrishnan V.R., Suh J.D. (2019). The Role of *Staphylococcus aureus* in Patients with Chronic Sinusitis and Nasal Polyposis. Curr. Allergy Asthma Rep..

[221] Teufelberger A.R., Bröker B.M., Krysko D.V., Bachert C., Krysko O. (2019). *Staphylococcus aureus* Orchestrates Type 2 Airway Diseases. Trends Mol. Med..

[222] Jorde I., Schreiber J., Stegemann-Koniszewski S. (2022). The Role of *Staphylococcus aureus* and Its Toxins in the Pathogenesis of Allergic Asthma. Int. J. Mol. Sci..

[223] Bachert C., Humbert M., Hanania N.A., Zhang N., Holgate S., Buhl R., Bröker B.M. (2020). *Staphylococcus aureus* and Its IgE-Inducing Enterotoxins in Asthma: Current Knowledge. Eur. Respir. J..

[224] Ceccarelli F., Perricone C., Olivieri G., Cipriano E., Spinelli F.R., Valesini G., Conti F. (2019). *Staphylococcus aureus* Nasal Carriage and Autoimmune Diseases: From Pathogenic Mechanisms to Disease Susceptibility and Phenotype. Int. J. Mol. Sci..

[225] Chen H., Zhang J., He Y., Lv Z., Liang Z., Chen J., Li P., Liu J., Yang H., Tao A. (2022). Exploring the Role of *Staphylococcus aureus* in Inflammatory Diseases. Toxins.

[226] DeVore S.B., Gonzalez T., Sherenian M.G., Herr A.B., Hershey G.K.K. (2020). On the Surface: Skin Microbial Exposure Contributes to Allergic Disease. Ann. Allergy Asthma Immunol..

[227] Gevaert E., Zhang N., Krysko O., Lan F., Holtappels G., De Ruyck N., Nauwynck H., Yousefi S., Simon H.-U., Bachert C. (2017). Extracellular Eosinophilic Traps in Association with *Staphylococcus aureus* at the Site of Epithelial Barrier Defects in Patients with Severe Airway Inflammation. J. Allergy Clin. Immunol..

[228] Gangwar R.S., Levi-Schaffer F. (2016). sCD48 Is Anti-Inflammatory in *Staphylococcus aureus* Enterotoxin B-Induced Eosinophilic Inflammation. Allergy.

[229] Minai-Fleminger Y., Gangwar R.S., Migalovich-Sheikhet H., Seaf M., Leibovici V., Hollander N., Feld M., Moses A.E., Homey B., Levi-Schaffer F. (2014). The CD48 Receptor Mediates *Staphylococcus aureus* Human and Murine Eosinophil Activation. Clin. Exp. Allergy.

[230] Leyva-Castillo J.-M., Das M., Kane J., Strakosha M., Singh S., Wong D.S.H., Horswill A.R., Karasuyama H., Brombacher F., Miller L.S. (2021). Basophil-Derived IL-4 Promotes Cutaneous *Staphylococcus aureus* Infection. JCI Insight.

[231] Bachert C., van Steen K., Zhang N., Holtappels G., Cattaert T., Maus B., Buhl R., Taube C., Korn S., Kowalski M. (2012). Specific IgE against *Staphylococcus aureus* Enterotoxins: An Independent Risk Factor for Asthma. J. Allergy Clin. Immunol..

[232] Kowalski M.L., Cieślak M., Pérez-Novo C.A., Makowska J.S., Bachert C. (2011). Clinical and Immunological Determinants of Severe/Refractory Asthma (SRA): Association with Staphylococcal Superantigen-Specific IgE Antibodies. Allergy.

[233] Haruna T., Kariya S., Fujiwara T., Higaki T., Makihara S., Kanai K., Fujiwara R., Iwasaki S., Noguchi Y., Nishizaki K. (2018). Association between Impaired IL-10 Production Following Exposure to *Staphylococcus aureus* Enterotoxin B and Disease Severity in Eosinophilic Chronic Rhinosinusitis. Allergol. Int..

[234] Hellings P.W., Hens G., Meyts I., Bullens D., Vanoirbeek J., Gevaert P., Jorissen M., Ceuppens J.L., Bachert C. (2006). Aggravation of Bronchial Eosinophilia in Mice by Nasal and Bronchial Exposure to *Staphylococcus aureus* Enterotoxin B. Clin. Exp. Allergy.

[235] Pastacaldi C., Lewis P., Howarth P. (2011). Staphylococci and Staphylococcal Superantigens in Asthma and Rhinitis: A Systematic Review and Meta-Analysis. Allergy.

[236] Caruso C., Colantuono S., Ciasca G., Basile U., Di Santo R., Bagnasco D., Passalacqua G., Caminati M., Michele S., Senna G. (2023). Different Aspects of Severe Asthma in Real Life: Role of *Staphylococcus aureus* Enterotoxins and Correlation to Comorbidities and Disease Severity. Allergy.

[237] Marone G., Tamburini M., Giudizi M.G., Biagiotti R., Almerigogna F., Romagnani S. (1987). Mechanism of Activation of Human Basophils by *Staphylococcus aureus* Cowan 1. Infect. Immun..

[238] Gaur P., Seaf M., Trabelsi N., Marcu O., Gafarov D., Schueler-Furman O., Mandelboim O., Ben-Zimra M., Levi-Schaffer F. (2024). 2B4: A Potential Target in *Staphylococcus aureus* Associated Allergic Inflammation. Clin. Exp. Immunol..

[239] Dunzendorfer S., Kaneider N.C., Kaser A., Woell E., Frade J.M.R., Mellado M., Martínez-Alonso C., Wiedermann C.J. (2001). Functional Expression of Chemokine Receptor 2 by Normal Human Eosinophils. J. Allergy Clin. Immunol..

[240] Suzukawa M., Koketsu R., Iikura M., Nakae S., Matsumoto K., Nagase H., Saito H., Matsushima K., Ohta K., Yamamoto K. (2008). Interleukin-33 Enhances Adhesion, CD11b Expression and Survival in Human Eosinophils. Lab. Investig..

[241] Blaylock M.G., Lipworth B.J., Dempsey O.J., Duncan C.J.A., Lee D.K.C., Lawrie A., Douglas J.G., Walsh G.M. (2003). Eosinophils from Patients with Asthma Express Higher Levels of the Pan-Leucocyte Receptor CD45 and the Isoform CD45RO. Clin. Exp. Allergy.

[242] Wechsler M.E., Munitz A., Ackerman S.J., Drake M.G., Jackson D.J., Wardlaw A.J., Dougan S.K., Berdnikovs S., Schleich F., Matucci A. (2021). Eosinophils in Health and Disease: A State-of-the-Art Review. Mayo Clin. Proc..

[243] Zwirner J., Götze O., Begemann G., Kapp A., Kirchhoff K., Werfel T. (1999). Evaluation of C3a Receptor Expression on Human Leucocytes by the Use of Novel Monoclonal Antibodies. Immunology.

[244] Emtenani S., Holtsche M.M., Stahlkopf R., Seiler D.L., Burn T., Liu H., Parker M., Yilmaz K., Dikmen H.O., Lang M.H. (2022). Differential Expression of C5aR1 and C5aR2 in Innate and Adaptive Immune Cells Located in Early Skin Lesions of Bullous Pemphigoid Patients. Front. Immunol..

[245] Steiner M., Huber S., Harrer A., Himly M. (2016). The Evolution of Human Basophil Biology from Neglect towards Understanding of Their Immune Functions. Biomed. Res. Int..

[246] Iikura M., Miyamasu M., Yamaguchi M., Kawasaki H., Matsushima K., Kitaura M., Morita Y., Yoshie O., Yamamoto K., Hirai K. (2001). Chemokine Receptors in Human Basophils: Inducible Expression of Functional CXCR4. J. Leukoc. Biol..

[247] Pan Q., Feng Y., Peng Y., Zhou H., Deng Z., Li L., Han H., Lin J., Shi L., Wang S. (2017). Basophil Recruitment to Skin Lesions of Patients with Systemic Lupus Erythematosus Mediated by CCR1 and CCR2. Cell. Physiol. Biochem..

[248] Bochner B.S., McKelvey A.A., Sterbinsky S.A., Hildreth J.E., Derse C.P., Klunk D.A., Lichtenstein L.M., Schleimer R.P. (1990). IL-3 Augments Adhesiveness for Endothelium and CD11b Expression in Human Basophils but Not Neutrophils. J. Immunol..

[249] Han X., Jorgensen J.L., Brahmandam A., Schlette E., Huh Y.O., Shi Y., Awagu S., Chen W. (2008). Immunophenotypic Study of Basophils by Multiparameter Flow Cytometry. Arch. Pathol. Lab. Med..

[250] Füreder W., Agis H., Willheim M., Bankl H.C., Maier U., Kishi K., Müller M.R., Czerwenka K., Radaszkiewicz T., Butterfield J.H. (1995). Differential Expression of Complement Receptors on Human Basophils and Mast Cells. Evidence for Mast Cell Heterogeneity and CD88/C5aR Expression on Skin Mast Cells. J. Immunol..

[251] Gane P., Pecquet C., Lambin P., Abuaf N., Leynadier F., Rouger P. (1993). Flow Cytometric Evaluation of Human Basophils. Cytometry.

